# Bis(nitrato-κ*O*)(5,7,12,14-tetra­methyl-1,4,8,11-tetra­aza­cyclo­tetra­decane-6,13-diaminium-κ^4^ *N* ^1^,*N* ^4^,*N* ^8^,*N* ^11^)copper(II) dinitrate tetra­hydrate

**DOI:** 10.1107/S1600536810023342

**Published:** 2010-06-23

**Authors:** Xiang-Yun Liu, Hong-Ying Chu

**Affiliations:** a Department of Chemistry and Chemical Engineering, Henan University of Urban Construction, Pingdingshan 467044, People’s Republic of China; b Department of Environment and Chemical Engineering, Yellow River Conservancy Technical Institute, Kaifeng 475004, People’s Republic of China

## Abstract

In the title compound, [Cu(NO_3_)_2_(C_14_H_36_N_6_)](NO_3_)_2_·4H_2_O, the Cu^II^ atom, lying on an inversion center, is six-coordinated in a distorted octa­hedral environment by four N atoms from a centrosymmetric 14-membered tetra­aza­cyclo­tetra­decane macrocyclic ligand and two O atoms from two nitrate anions. The supra­molecular network is consolidated by extensive O—H⋯O and N—H⋯O hydrogen-bonding inter­actions.

## Related literature

For Cu(II) complexes of related macrocyclic ligands, see: Bernhardt (1999 ▶); Bernhardt & Sharpe (1998 ▶).
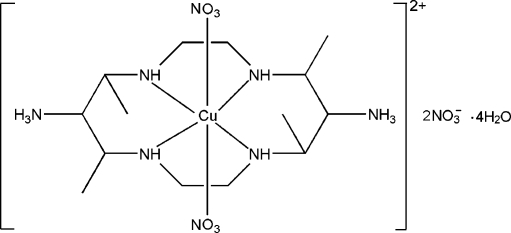


## Experimental

### 

#### Crystal data

[Cu(NO_3_)_2_(C_14_H_36_N_6_)](NO_3_)_2_·4H_2_O
*M* *_r_* = 672.14
Monoclinic, 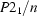

*a* = 9.201 (2) Å
*b* = 16.576 (4) Å
*c* = 9.278 (2) Å
β = 98.788 (4)°
*V* = 1398.4 (5) Å^3^
*Z* = 2
Mo *K*α radiation
μ = 0.87 mm^−1^
*T* = 123 K
0.37 × 0.34 × 0.31 mm

#### Data collection

Bruker SMART 1000 CCD diffractometer
Absorption correction: multi-scan (*SADABS*; Bruker, 2001 ▶) *T*_min_ = 0.739, *T*_max_ = 0.774
6071 measured reflections
3021 independent reflections
2269 reflections with *I* > 2σ(*I*)
*R*_int_ = 0.027

#### Refinement

*R*[*F* ^2^ > 2σ(*F* ^2^)] = 0.046
*wR*(*F* ^2^) = 0.133
*S* = 1.03
3021 reflections
202 parameters
6 restraints
H atoms treated by a mixture of independent and constrained refinement
Δρ_max_ = 1.24 e Å^−3^
Δρ_min_ = −0.81 e Å^−3^

### 

Data collection: *SMART* (Bruker, 2007 ▶); cell refinement: *SAINT* (Bruker, 2007 ▶); data reduction: *SAINT*; program(s) used to solve structure: *SHELXS97* (Sheldrick, 2008 ▶); program(s) used to refine structure: *SHELXL97* (Sheldrick, 2008 ▶); molecular graphics: *SHELXTL* (Sheldrick, 2008 ▶); software used to prepare material for publication: *SHELXTL*.

## Supplementary Material

Crystal structure: contains datablocks I, global. DOI: 10.1107/S1600536810023342/hy2316sup1.cif

Structure factors: contains datablocks I. DOI: 10.1107/S1600536810023342/hy2316Isup2.hkl

Additional supplementary materials:  crystallographic information; 3D view; checkCIF report

## Figures and Tables

**Table 1:** Hydrogen-bond geometry (Å, °)

*D*—H⋯*A*	*D*—H	H⋯*A*	*D*⋯*A*	*D*—H⋯*A*
N1—H1*C*⋯O3	0.93	2.43	3.155 (4)	134
N1—H1*C*⋯O2*W*^i^	0.93	2.28	3.096 (4)	146
N2—H2*A*⋯O3	0.93	2.52	3.244 (4)	135
N2—H2*A*⋯O4^ii^	0.93	2.47	3.249 (4)	141
N3—H3*D*⋯O4	0.91	2.08	2.924 (4)	155
N3—H3*E*⋯O1*W*^iii^	0.91	1.86	2.748 (4)	164
N3—H3*F*⋯O2^i^	0.91	2.06	2.902 (4)	154
N3—H3*F*⋯O3^i^	0.91	2.32	3.108 (4)	145
O1*W*—H1*WA*⋯O2^i^	0.84 (4)	2.01 (3)	2.823 (4)	160 (5)
O1*W*—H1*WB*⋯O5^iv^	0.84 (2)	1.97 (2)	2.795 (4)	168 (5)
O2*W*—H2*WA*⋯O6^ii^	0.93 (5)	2.00 (5)	2.913 (5)	166 (5)
O2*W*—H2*WB*⋯O6^v^	0.92 (2)	2.18 (2)	3.084 (5)	167 (5)

Symmetry codes: (i) 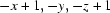; (ii) 
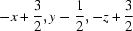
; (iii) 
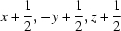
; (iv) 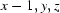; (v) 
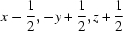
.

**Fig. 1.**
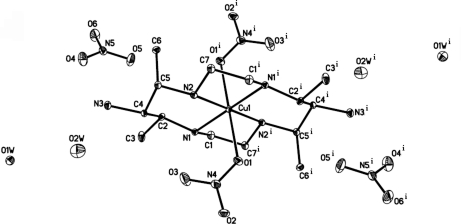
The molecular structure of the title compound. Displacement ellipsoids are shown at the 30% probability level. H atoms have been omitted for clarity. [Symmetry code: (i) 2-x, -y, 1-z.]

## supplementary crystallographic information

### Comment

In the past, much attention has been given to the copper complexes of macrocyclic *trans*-5(*R*),7(*R*),12(*R*), 14(*R*)-tetramethyl-6, 13-dinitro-1,4,8,11-tetraazacyclotetradecane and related ligands (Bernhardt, 1999; Bernhardt & Sharpe, 1998). Recently, we have synthesized a Cu(II) complex based on 5,7,12,14-tetramethyl-6,13- diamino-1,4,8,11-tetraazacyclotetradecane and its structure is reported here.

The asymmetric unit of the title compound (Fig. 1) contains one Cu^II^ ion lying on an inversion center, one half of a 14-membered tetraazacyclotetradecane macrocyclic ligand, one coordinated nitrate anion, one uncoordinated nitrate anion and two solvent water molecules. The Cu^II^ ion has a slightly distorted octahedral coordination geometry, with two O atoms from two nitrate anions in the axial positions. The equatorial positions are occupied by four N atoms from the centrosymmetric 14-membered tetraazacyclotetradecane macrocyclic ligand [Cu1—N1 2.025 (2) and Cu1—N2 2.020 (2) Å]. The two uncoordinated nitrate anions are located above and below the 14-membered tetraazacyclotetradecane macrocycle and linked to the macrocycle *via* N—H···O hydrogen bonds (Table 1).

### Experimental

An aqueous solution of 5,7,12,14-tetramethyl-6,13-diamino-1,4,8,11-tetraazacyclotetradecane (0.27 g, 1.0 mmol), Cu(NO_3_)_2_ (0.10 g, 0.5 mmol) and Na_2_CO_3_ (0.05 g, 0.5 mmol) was heated to reflux for 24 h. The reaction mixture was cooled to room temperature and red crystals of the title compound were obtained by slow evaporation of the solvent at room temperature.

### Refinement

H atoms bound to C and N atoms were placed at calculated positions and were treated as riding on the parent atoms, with C—H = 1.00 (CH), 0.99 (CH_2_) and 0.98 (CH_3_) Å and N—H = 0.93 (NH) and 0.91 (NH_3_) Å and with *U*_iso_(H) = 1.2–1.5 *U*_eq_(C, N). H atoms attached to water molecules were located in a difference Fourier map and refined with *U*_iso_(H) = 1.2*U*_eq_(O). The highest residual electron density was found 0.91 Å from O3 the deepest hole 0.52 Å from H2WA.

### Crystal data

**Table:**

[Cu(NO_3_)_2_(C_14_H_36_N_6_)](NO_3_)_2_·4H_2_O	*F*(000) = 710
*M**_r_* = 672.14	*D*_x_ = 1.596 Mg m^−^^3^
Monoclinic, *P*2_1_/*n*	Mo *K*α radiation, λ = 0.71073 Å
Hall symbol: -P 2yn	Cell parameters from 2543 reflections
*a* = 9.201 (2) Å	θ = 2.5–27.0°
*b* = 16.576 (4) Å	µ = 0.87 mm^−^^1^
*c* = 9.278 (2) Å	*T* = 123 K
β = 98.788 (4)°	Block, red
*V* = 1398.4 (5) Å^3^	0.37 × 0.34 × 0.31 mm
*Z* = 2	

### Data collection

**Table:**

Bruker SMART 1000 CCD diffractometer	3021 independent reflections
Radiation source: fine-focus sealed tube	2269 reflections with *I* > 2σ(*I*)
graphite	*R*_int_ = 0.027
φ and ω scans	θ_max_ = 27.1°, θ_min_ = 2.5°
Absorption correction: multi-scan (*SADABS*; Bruker, 2001)	*h* = −11→11
*T*_min_ = 0.739, *T*_max_ = 0.774	*k* = −21→17
6071 measured reflections	*l* = −10→11

### Refinement

**Table:**

Refinement on *F*^2^	Primary atom site location: structure-invariant direct methods
Least-squares matrix: full	Secondary atom site location: difference Fourier map
*R*[*F*^2^ > 2σ(*F*^2^)] = 0.046	Hydrogen site location: inferred from neighbouring sites
*wR*(*F*^2^) = 0.133	H atoms treated by a mixture of independent and constrained refinement
*S* = 1.03	*w* = 1/[σ^2^(*F*_o_^2^) + (0.0649*P*)^2^ + 2.4391*P*] where *P* = (*F*_o_^2^ + 2*F*_c_^2^)/3
3021 reflections	(Δ/σ)_max_ = 0.036
202 parameters	Δρ_max_ = 1.24 e Å^−^^3^
6 restraints	Δρ_min_ = −0.81 e Å^−^^3^

### Fractional atomic coordinates and isotropic or equivalent isotropic displacement parameters (Å^2^)

**Table:**

	*x*	*y*	*z*	*U*_iso_*/*U*_eq_	
C1	0.8753 (4)	0.0883 (2)	0.2521 (3)	0.0169 (7)	
H1A	0.9413	0.1356	0.2695	0.020*	
H1B	0.7886	0.1039	0.1807	0.020*	
C2	0.7676 (3)	0.13149 (18)	0.4684 (3)	0.0136 (6)	
H2	0.8477	0.1722	0.4930	0.016*	
C3	0.6400 (4)	0.1717 (2)	0.3687 (3)	0.0234 (8)	
H3A	0.6749	0.1921	0.2809	0.035*	
H3B	0.6019	0.2167	0.4205	0.035*	
H3C	0.5616	0.1322	0.3408	0.035*	
C4	0.7205 (3)	0.10160 (19)	0.6114 (3)	0.0129 (6)	
H4	0.6574	0.0527	0.5888	0.016*	
C5	0.8448 (3)	0.08013 (19)	0.7366 (3)	0.0137 (6)	
H5	0.7973	0.0642	0.8224	0.016*	
C6	0.9474 (3)	0.15076 (19)	0.7843 (3)	0.0167 (7)	
H6A	1.0086	0.1378	0.8774	0.025*	
H6B	0.8889	0.1991	0.7960	0.025*	
H6C	1.0105	0.1608	0.7102	0.025*	
C7	1.0453 (3)	−0.0192 (2)	0.8066 (3)	0.0170 (7)	
H7A	1.0050	−0.0366	0.8947	0.020*	
H7B	1.1144	0.0259	0.8342	0.020*	
Cu1	1.0000	0.0000	0.5000	0.01124 (16)	
N1	0.8274 (3)	0.06223 (16)	0.3922 (2)	0.0120 (5)	
H1C	0.7509	0.0256	0.3683	0.014*	
N2	0.9236 (3)	0.00785 (15)	0.6924 (3)	0.0128 (5)	
H2A	0.8545	−0.0334	0.6882	0.015*	
N3	0.6297 (3)	0.16570 (17)	0.6691 (3)	0.0173 (6)	
H3D	0.6722	0.2147	0.6611	0.026*	
H3E	0.6241	0.1555	0.7645	0.026*	
H3F	0.5376	0.1657	0.6167	0.026*	
N5	0.7860 (3)	0.36290 (18)	0.6169 (3)	0.0214 (6)	
O4	0.6922 (3)	0.33861 (18)	0.6897 (3)	0.0377 (7)	
O5	0.8513 (3)	0.31313 (17)	0.5511 (3)	0.0399 (7)	
O6	0.8117 (3)	0.43623 (16)	0.6061 (4)	0.0402 (7)	
O1W	0.1266 (3)	0.33514 (16)	0.4647 (3)	0.0236 (5)	
O2W	0.4173 (4)	0.0272 (3)	0.8131 (4)	0.0592 (10)	
H1WA	0.168 (5)	0.291 (2)	0.489 (6)	0.071*	
H1WB	0.039 (3)	0.334 (3)	0.481 (6)	0.071*	
H2WA	0.494 (5)	−0.008 (3)	0.846 (6)	0.071*	
H2WB	0.396 (6)	0.045 (3)	0.901 (3)	0.071*	
N4	0.7300 (3)	−0.14301 (18)	0.4733 (3)	0.0233 (6)	
O1	0.8658 (2)	−0.13506 (15)	0.4843 (2)	0.0226 (5)	
O2	0.6711 (3)	−0.21048 (15)	0.4441 (3)	0.0252 (6)	
O3	0.6522 (3)	−0.08487 (18)	0.4963 (4)	0.0520 (9)	

### Atomic displacement parameters (Å^2^)

**Table:**

	*U*^11^	*U*^22^	*U*^33^	*U*^12^	*U*^13^	*U*^23^
C1	0.0194 (16)	0.0188 (17)	0.0124 (14)	0.0044 (13)	0.0023 (11)	0.0043 (12)
C2	0.0175 (15)	0.0104 (15)	0.0122 (14)	0.0038 (12)	0.0004 (11)	0.0000 (11)
C3	0.0233 (18)	0.032 (2)	0.0150 (15)	0.0136 (15)	0.0039 (13)	0.0031 (14)
C4	0.0113 (14)	0.0153 (16)	0.0120 (13)	0.0026 (12)	0.0014 (11)	−0.0013 (11)
C5	0.0141 (15)	0.0155 (16)	0.0117 (13)	0.0025 (12)	0.0021 (11)	0.0011 (11)
C6	0.0171 (15)	0.0130 (16)	0.0192 (15)	0.0000 (12)	−0.0003 (12)	−0.0031 (12)
C7	0.0175 (15)	0.0232 (18)	0.0094 (13)	0.0068 (13)	−0.0009 (11)	0.0019 (11)
Cu1	0.0120 (3)	0.0135 (3)	0.0079 (2)	0.0030 (2)	0.00051 (17)	0.0002 (2)
N1	0.0121 (12)	0.0128 (13)	0.0112 (11)	0.0008 (10)	0.0022 (9)	−0.0003 (10)
N2	0.0123 (12)	0.0143 (14)	0.0113 (11)	0.0017 (10)	−0.0001 (9)	0.0009 (10)
N3	0.0161 (13)	0.0226 (16)	0.0134 (12)	0.0050 (11)	0.0030 (10)	−0.0006 (11)
N5	0.0167 (14)	0.0227 (16)	0.0244 (14)	0.0033 (12)	0.0021 (11)	0.0008 (12)
O4	0.0357 (16)	0.0389 (17)	0.0442 (16)	0.0024 (13)	0.0241 (13)	0.0093 (13)
O5	0.0442 (17)	0.0260 (15)	0.0558 (18)	−0.0001 (13)	0.0282 (14)	−0.0111 (13)
O6	0.0382 (16)	0.0149 (14)	0.071 (2)	−0.0009 (12)	0.0184 (14)	−0.0028 (13)
O1W	0.0252 (13)	0.0268 (14)	0.0192 (12)	−0.0001 (11)	0.0047 (10)	0.0005 (10)
O2W	0.051 (2)	0.068 (3)	0.056 (2)	0.0019 (19)	−0.0006 (17)	−0.0158 (19)
N4	0.0187 (14)	0.0206 (16)	0.0305 (16)	−0.0036 (12)	0.0031 (12)	−0.0016 (12)
O1	0.0153 (11)	0.0264 (14)	0.0268 (12)	−0.0057 (10)	0.0053 (9)	−0.0017 (10)
O2	0.0212 (12)	0.0197 (13)	0.0327 (13)	−0.0060 (10)	−0.0022 (10)	−0.0022 (10)
O3	0.0230 (15)	0.0230 (16)	0.111 (3)	0.0001 (12)	0.0151 (16)	−0.0074 (17)

### Geometric parameters (Å, °)

**Table:**

C1—N1	1.499 (4)	C7—C1^i^	1.505 (4)
C1—C7^i^	1.505 (4)	C7—H7A	0.9900
C1—H1A	0.9900	C7—H7B	0.9900
C1—H1B	0.9900	Cu1—N2	2.020 (2)
C2—N1	1.496 (4)	Cu1—N1	2.025 (2)
C2—C3	1.532 (4)	Cu1—O1	2.550 (2)
C2—C4	1.539 (4)	N1—H1C	0.9300
C2—H2	1.0000	N2—H2A	0.9300
C3—H3A	0.9800	N3—H3D	0.9100
C3—H3B	0.9800	N3—H3E	0.9100
C3—H3C	0.9800	N3—H3F	0.9100
C4—N3	1.500 (4)	N5—O5	1.235 (4)
C4—C5	1.542 (4)	N5—O4	1.241 (4)
C4—H4	1.0000	N5—O6	1.245 (4)
C5—N2	1.491 (4)	O1W—H1WA	0.84 (4)
C5—C6	1.526 (4)	O1W—H1WB	0.84 (2)
C5—H5	1.0000	O2W—H2WA	0.93 (5)
C6—H6A	0.9800	O2W—H2WB	0.91 (2)
C6—H6B	0.9800	N4—O3	1.239 (4)
C6—H6C	0.9800	N4—O1	1.245 (4)
C7—N2	1.488 (3)	N4—O2	1.254 (4)
			
N1—C1—C7^i^	108.5 (2)	N2—C7—H7A	109.9
N1—C1—H1A	110.0	C1^i^—C7—H7A	109.9
C7^i^—C1—H1A	110.0	N2—C7—H7B	109.9
N1—C1—H1B	110.0	C1^i^—C7—H7B	109.9
C7^i^—C1—H1B	110.0	H7A—C7—H7B	108.3
H1A—C1—H1B	108.4	N2^i^—Cu1—N1	87.06 (10)
N1—C2—C3	110.6 (2)	N2—Cu1—N1	92.94 (10)
N1—C2—C4	109.4 (2)	O1—Cu1—N1	94.74 (9)
C3—C2—C4	111.7 (3)	O1—Cu1—N2	82.89 (8)
N1—C2—H2	108.3	O1—Cu1—N1^i^	85.26 (9)
C3—C2—H2	108.3	O1—Cu1—N2^i^	97.11 (8)
C4—C2—H2	108.3	C2—N1—C1	111.5 (2)
C2—C3—H3A	109.5	C2—N1—Cu1	118.36 (17)
C2—C3—H3B	109.5	C1—N1—Cu1	105.27 (18)
H3A—C3—H3B	109.5	C2—N1—H1C	107.1
C2—C3—H3C	109.5	C1—N1—H1C	107.1
H3A—C3—H3C	109.5	Cu1—N1—H1C	107.1
H3B—C3—H3C	109.5	C7—N2—C5	113.0 (2)
N3—C4—C2	109.0 (2)	C7—N2—Cu1	106.55 (18)
N3—C4—C5	106.5 (2)	C5—N2—Cu1	123.02 (18)
C2—C4—C5	116.8 (3)	C7—N2—H2A	104.1
N3—C4—H4	108.1	C5—N2—H2A	104.1
C2—C4—H4	108.1	Cu1—N2—H2A	104.1
C5—C4—H4	108.1	C4—N3—H3D	109.5
N2—C5—C6	113.0 (2)	C4—N3—H3E	109.5
N2—C5—C4	108.3 (2)	H3D—N3—H3E	109.5
C6—C5—C4	113.3 (3)	C4—N3—H3F	109.5
N2—C5—H5	107.3	H3D—N3—H3F	109.5
C6—C5—H5	107.3	H3E—N3—H3F	109.5
C4—C5—H5	107.3	O5—N5—O4	118.9 (3)
C5—C6—H6A	109.5	O5—N5—O6	120.0 (3)
C5—C6—H6B	109.5	O4—N5—O6	121.1 (3)
H6A—C6—H6B	109.5	H1WA—O1W—H1WB	109 (4)
C5—C6—H6C	109.5	H2WA—O2W—H2WB	99 (4)
H6A—C6—H6C	109.5	O3—N4—O1	120.2 (3)
H6B—C6—H6C	109.5	O3—N4—O2	119.3 (3)
N2—C7—C1^i^	109.0 (2)	O1—N4—O2	120.5 (3)
			
N1—C2—C4—N3	−167.0 (2)	N2^i^—Cu1—N1—C2	−142.1 (2)
C3—C2—C4—N3	−44.1 (3)	N2—Cu1—N1—C2	37.9 (2)
N1—C2—C4—C5	72.4 (3)	N2^i^—Cu1—N1—C1	−16.78 (19)
C3—C2—C4—C5	−164.7 (3)	N2—Cu1—N1—C1	163.22 (19)
N3—C4—C5—N2	171.2 (2)	C1^i^—C7—N2—C5	−175.9 (3)
C2—C4—C5—N2	−66.9 (3)	C1^i^—C7—N2—Cu1	−37.9 (3)
N3—C4—C5—C6	−62.6 (3)	C6—C5—N2—C7	54.5 (3)
C2—C4—C5—C6	59.3 (3)	C4—C5—N2—C7	−179.2 (2)
C3—C2—N1—C1	56.7 (3)	C6—C5—N2—Cu1	−75.7 (3)
C4—C2—N1—C1	−179.9 (2)	C4—C5—N2—Cu1	50.7 (3)
C3—C2—N1—Cu1	178.9 (2)	N1^i^—Cu1—N2—C7	11.4 (2)
C4—C2—N1—Cu1	−57.6 (3)	N1—Cu1—N2—C7	−168.6 (2)
C7^i^—C1—N1—C2	171.5 (2)	N1^i^—Cu1—N2—C5	144.2 (2)
C7^i^—C1—N1—Cu1	42.0 (3)	N1—Cu1—N2—C5	−35.8 (2)

Symmetry codes: (i) −*x*+2, −*y*, −*z*+1.

### Hydrogen-bond geometry (Å, °)

**Table:**

*D*—H···*A*	*D*—H	H···*A*	*D*···*A*	*D*—H···*A*
N1—H1C···O3	0.93	2.43	3.155 (4)	134
N1—H1C···O2W^ii^	0.93	2.28	3.096 (4)	146
N2—H2A···O3	0.93	2.52	3.244 (4)	135
N2—H2A···O4^iii^	0.93	2.47	3.249 (4)	141
N3—H3D···O4	0.91	2.08	2.924 (4)	155
N3—H3E···O1W^iv^	0.91	1.86	2.748 (4)	164
N3—H3F···O2^ii^	0.91	2.06	2.902 (4)	154
N3—H3F···O3^ii^	0.91	2.32	3.108 (4)	145
O1W—H1WA···O2^ii^	0.84 (4)	2.01 (3)	2.823 (4)	160 (5)
O1W—H1WB···O5^v^	0.84 (2)	1.97 (2)	2.795 (4)	168 (5)
O2W—H2WA···O6^iii^	0.93 (5)	2.00 (5)	2.913 (5)	166 (5)
O2W—H2WB···O6^vi^	0.92 (2)	2.18 (2)	3.084 (5)	167 (5)

Symmetry codes: (ii) −*x*+1, −*y*, −*z*+1; (iii) −*x*+3/2, *y*−1/2, −*z*+3/2; (iv) *x*+1/2, −*y*+1/2, *z*+1/2; (v) *x*−1, *y*, *z*; (vi) *x*−1/2, −*y*+1/2, *z*+1/2.
